# Knowledge and perceptions of Indian primary care nurses towards mental illness

**DOI:** 10.17533/udea.iee.v37n1a06

**Published:** 2019-01-06

**Authors:** Sailaxmi Gandhi, Vijayalakshmi Poreddi, Radhakrishnan Govindan, Jothimani G, Shamala Anjanappa, Maya Sahu, Padmavathi Narayanasamy, Manjunath N, Naveenkumar C, Suresh Badamath

**Affiliations:** 1 RN, RM, BSN, MSN, Ph.D, Additional Profesor & Head, Department of Nursing, National Institute of Mental Health & Neurosciences (Indian Institute of National Importance) India. email: sailaxmi63@yahoo.com National Institute of Mental Health & Neurosciences (Indian Institute of National Importance) India sailaxmi63@yahoo.com; 2 RN, RM, BSN, MSN. PhD Scholar, Department of Nursing, National Institute of Mental Health & Neurosciences (Indian Institute of National Importance) India. email: pvijayalakshmireddy@gmail.com. Corresponding author National Institute of Mental Health & Neurosciences (Indian Institute of National Importance) India pvijayalakshmireddy@gmail.com; 3 RN, RM, BSN, MSN. PhD, Assistant Professor, Department of Nursing, National Institute of Mental Health & Neurosciences (Indian Institute of National Importance) India. email: dr.rk76@hotmail.com National Institute of Mental Health & Neurosciences (Indian Institute of National Importance) India dr.rk76@hotmail.com; 4 RN, RM, BSN, MSN. PhD Scholar, Department of Nursing, National Institute of Mental Health & Neurosciences (Indian Institute of National Importance), India. email: jothisrinivas.jothi@gmail.com National Institute of Mental Health & Neurosciences (Indian Institute of National Importance) India jothisrinivas.jothi@gmail.com; 5 RN, RM, BSN, MSN. PhD Scholar, Department of Nursing, National Institute of Mental Health & Neurosciences (Indian Institute of National Importance), India. email: kushraghusham@gmail.com National Institute of Mental Health & Neurosciences (Indian Institute of National Importance) India kushraghusham@gmail.com; 6 RN, RM, BSN, MSN. Ph.D Scholar, Department of Nursing, National Institute of Mental Health & Neurosciences (Indian Institute of National Importance), India. email: mayamonsahu@gmail.com National Institute of Mental Health & Neurosciences (Indian Institute of National Importance) India mayamonsahu@gmail.com; 7 RN, RM, BSN, MSN. Ph.D Scholar, Department of Nursing, National Institute of Mental Health & Neurosciences (Indian Institute of National Importance), India. email: padmavathinarayanasamy@gmail.com National Institute of Mental Health & Neurosciences (Indian Institute of National Importance) India padmavathinarayanasamy@gmail.com; 8 MBBS,DPM, MD. Associate professor, Department of Psychiatry, National Institute of Mental Health & Neurosciences (Indian Institute of National Importance) India. email: manjunatha.adc@gmail.com National Institute of Mental Health & Neurosciences (Indian Institute of National Importance) India manjunatha.adc@gmail.com; 9 MBBS, MD. Additional professor, Department of Psychiatry, National Institute of Mental Health & Neurosciences (Indian Institute of National Importance), India. email: cnkumar1974@gmail.com National Institute of Mental Health & Neurosciences (Indian Institute of National Importance) India cnkumar1974@gmail.com; 10 MD, DNB, PGDMLE, PGDHRL, PhD in Law (NLSIU), MBBS, DPM, MD. Pofessor,Department of Psychiatry, National Institute of Mental Health & Neurosciences (Indian Institute of National Importance) India. email: nimhans@gmail.com National Institute of Mental Health & Neurosciences (Indian Institute of National Importance) India nimhans@gmail.com

**Keywords:** mentally ill persons, stereotyping, beneficence, optimism, pessimism, attitude, primary care nursing, cross-sectional studies, self-report., enfermos mentales, estereotipo, beneficencia, optimismo, pesimismo, actitud, enfermería de atención primaria, estudios transversales, autoinforme., pessoas mentalmente doentes, estereotipagem, beneficencia, otimismo, pesimismo, atitude, enfermagem de atenção primária, estudos transversais, autorrelato.

## Abstract

**Objective.:**

To assess nurses’ knowledge and perceptions towards mental illness.

**Methods.:**

This was a cross-sectional descriptive study conducted among 126 randomly selected nurses those are working under District Mental Health program in Karnataka (India). The data was collected through self-reported questionnaires Using the modified version of *Public perception of mental illness questionnaire* and *Attitude Scale for Mental Illness*.

**Results.:**

The findings revealed that majority of the subjects were women (74.4%), Hindus (92.1%) and were from rural background (69.8%). The mean Knowledge score 10.8±1.6 adequate knowledge (maximum possible =12) among 91% of the subjects, and 52% of them hold negative attitudes towards people with mental illness (88.9±13.6). While majority of the subjects hold negative attitudes in ‘Separatism’ (53.5%), ‘Stereotyping’ (73%), ‘Benevolence’ (54%), ‘Pessimistic prediction’ (53%) domains, they hold positive attitudes in ‘Restrictiveness’ (88%) and ‘Stigmatization’ (72%) domains. Women than men endorsed positive attitudes towards persons with mental illness in Stereotyping’ (*p*<0.001), ‘Restrictiveness’ (*p*<0.01), ‘Benevolence’ (*p*<0.001) and ‘Pessimistic prediction’ (t= 2.22, p<0.05) domains. Similarly, Auxiliary Nursing Midwifery found to be less restrictive (*p*<0.05), more benevolent (*p*<0.001) and less pessimistic (*p*<0.05) compared to nurses with higher education (General Nursing and Midwifery and Bachelor of Science in Nursing).

**Conclusion.:**

The present study showed adequate knowledge on mental illness among nurses. Yet they hold stigmatizing and negative attitudes towards mental illness. Hence, it is an urgent priority to develop and implement educational programs to inculcate positive attitudes towards people with mental illness to provide optimal care to this vulnerable population.

## Resumen

**Objetivo.:**

Evaluar los conocimientos y percepciones de las enfermeras de atención primaria hacia la enfermedad mental.

**Métodos.:**

Se realizó un estudio descriptivo transversal con 126 enfermeras que trabajan en el programa de Salud Mental del Distrito en Karnataka (India). Los datos se recolectaron a partir de cuestionarios contestados por autorreporte, empleando la versión modificada del cuestionario *Percepción pública de la enfermedad mental* y la Escala de Actitudes hacia la enfemedad mental.

**Resultados.:**

Los hallazgos revelaron que la mayoría de los participantes eran mujeres (74.4%), hindúes (92.1%) y de origen rural (69.8%). El puntaje promedio de conocimiento fue de 10.8±1.6, que indica un conocimiento adecuado (máximo posible = 12). El 52% de los participantes tienen actitudes negativas hacia las personas con enfermedades mentales, siendo mayor este porcentaje en los dominios 'Estereotipos' (73%), 'Benevolencia' (54%), 'Separatismo' (53.5%), 'Predicción pesimista' (53%); mientras que tienen actitudes positivas en 'Restricción' (88%) y 'Estigmatización '(72%). Las mujeres, en mayor porcentaje que los hombres, tuvieron actitudes positivas hacia las personas con enfermedades mentales en los dominios 'Estereotipos' (p<0.001), 'Restricción' (*p*<0.01), 'Benevolencia' (*p*<0.001) y 'Predicción pesimista' (*p*<0.05). Del mismo modo, las enfermeras parteras auxiliares fueron menos restrictivas (*p*<0.05), más benévolas (*p*<0.001) y menos pesimista (*p*<0.05) en comparación con las enfermeras con mayor educación (enfermeras parteras generales y licenciadas en ciencias de la enfermería).

**Conclusión.:**

El presente estudio mostró un conocimiento adecuado sobre las enfermedades mentales entre las enfermeras. Sin embargo, tienen actitudes estigmatizantes y negativas hacia la enfermedad mental. Por lo tanto, es una prioridad implementar programas educativos para la adquisición de actitudes positivas hacia las personas con enfermedades mentales con el fin de brindar una atención integral a esta población vulnerable.

## Resumo

**Objetivo.:**

Avaliar os conhecimentos e percepções à doença mental das enfermeiras de atenção primária.

**Métodos.:**

Se realizou um estudo descritivo transversal com 126 enfermeiras que trabalham no programa de Saúde Mental do Distrito em Karnataka (Índia). Os dados foram recolhidos a través de questionários respondidos por autorreporte, empregando a versão modificada do questionário *Percepção pública da doença mental* e a Escala de Atitudes à doença mental.

**Resultados.:**

As descobertas revelaram que a maioria dos participantes eram mulheres (74.4%), hindus (92.1%) e de origem rural (69.8%). A pontuação média de conhecimento foi de 10.8±1.6 indicando conhecimento adequado (máximo possível = 12). 52% dos participantes têm atitudes negativas às pessoas com doenças mentais, sendo maior esta porcentagem nos domínios 'Estereótipos' (73%), 'Benevolência' (54%), 'Separatismo' (53.5%), 'Predição pessimista' (53%); enquanto que têm atitudes positivas em 'Restrição' (88%) e 'Estigmas'(72%). As mulheres, em maior porcentagem que os homens, tiveram atitudes positivas às pessoas com doenças mentais nos domínios 'Estereótipos' (p<0.001), 'Restrição' (*p*<0.01), 'Benevolência' (*p*<0.001) e 'Predição pessimista' (*p*<0.05). Do mesmo modo, as enfermeiras parteiras auxiliares formam menos restritivas (*p*<0.05), mais benévolas (*p*<0.001) e menos pessimista (*p*<0.05) em comparação com as enfermeiras com maior educação (enfermeiras parteiras gerais e licenciadas em ciências da enfermagem).

**Conclusão.:**

O presente estudo mostrou um conhecimento adequado sobre as doenças mentais entre as enfermeiras. Porém, tem atitudes de estigmas e negativas à doença mental. Por tanto, é uma prioridade implementar programas educativos para o melhoramento das atitudes positivas às pessoas com doenças mentais com o fim de brindar uma atenção integral a esta população vulnerável.

## Introduction

Mental health is an urgent concern in India as every sixth Indian needs mental health help as reported by a recent National Mental Health Survey.(1) Further, while 20% of Indians suffer from a mental illness, only 10-12% of them seek help from mental health professionals mainly due to ignorance, stigma and discrimination that largely prevail in Indian community. The survey also found a huge treatment gap for various mental disorders ranged between 70% and 92%. District Mental Health Programme (DMHP) was initiated by India to ensure accessibility of mental health services at the community level by integrating mental health with the primary health services. Under this program, nurses working at primary care centers are trained to detect and refer the individuals with mental health problems. Nurses comprise the chief workforce in the Indian health care system. They also play an important role not only in recovery of people with mental illness, but also support their reintegration in to the community. Surprisingly, health care professionals hold negative and stigmatizing attitudes towards mental illness to an extent that can be comparable with generable population.(2) An earlier research reported that the predictors for nurses having negative and unfavourable attitudes towards mental illness include; lack of knowledge,(3) lower education,(4) less professional experience, and not having any contact with persons with mental illness, etc.(5) 

In India, 90% of the people with mental illness live with their families.(6) Moreover, in a recent national survey conducted across eight cities of the country by ‘*The Live Love Laugh Foundation’* (n=3 556) reported that public had adequate knowledge awareness on mental illness (87%). Yet, they possessed high level of stigma towards people with mental illness as they used pejorative words such as *‘retard’* (47%) or ‘crazy’ ‘mad’ ‘stupid’ (40%) or ‘careless’ ‘irresponsible’ (38%) to describe people with mental illness.(7) Nurses and Auxiliary Nurse Midwives under DMHP program serve people who live in rural areas. Nurses being primary health care providers play a key role in combating against stigma and discrimination against people with mental illness as they provide care to the patients and are also involved in disseminating health information to the family members.(8) Further, in India most of the research focused on understanding attitudes among nursing and medical undergraduate students.(9-12) To date there is limited research on nurses' attitudes towards mental illness in Indian settings.(13) Hence, it is critical to assess knowledge and attitudes of nurses those are working at primary health‐care settings. In the present study, attitude refers to nurses’ emotional, cognitive, and behavioral responses to persons with mental illness.(14) 

## Methods

This was a cross sectional descriptive survey carried out among DMHP nurses who participated in a training program conducted by the Departments of Nursing and Community Psychiatry at National Institute of Mental Health and Neuro Sciences (NIMHANS) in the year 2017. 

Sample. The sample for the present study was selected using random sampling technique. The nurses who were working under District Mental Health Program were randomly selected from various districts of Karnataka State, to undergo training in an onsite program held at National Institute of Mental Health neurosciences, Bangalore, India. The data was collected from the subjects prior to the training program. The study criteria include a) Registered nurse or Midwife b) working in District Mental Health program or rural health program. The questionnaires were distributed to 162 nurses. Only 146 completed questionnaires were received. However, uncompleted questionnaires (*n*=16) were discarded and the final sample comprised of 126 with 77.8% response rate.

**Data collection Instruments. 1-*Demographic data survey instrument:*** The demographic form comprised of eight items to obtain the background information of the subjects in the study that included; age, gender, religion, professional qualification, residence, source of information and level of interest to learn about mental illness. **2-*Knowledge assessment questionnaire:*** Modified version of ‘***Public Perception of Mental Illness Questionnaire’*** (15) was used to collect subjects awareness on the risk factors for development of mental illness. This part of the tool consisted of 12 items with three responses namely ‘Yes’, ‘No’ and ‘Don’t Know’. One mark was awarded for every correct response, zero otherwise. Hence, the total score for knowledge questionnaire ranged from 0 to 12. **3-*Attitude scale for mental illness -ASMI-:*** This was a valid and reliable (Cronbach’s Alpha 0.86), self-report measure used to measure health professionals attitudes toward persons with mental illness.(16) This was a 5‑point Likert scale rated subjects’ responses from totally disagree (1) to totally agree (5). The lower scores indicate positive attitudes toward persons with mental illness. This tool has six sub scales: **(i) Separatism:** Ten items, (1-9, and 24) to measure respondents’ attitude of discrimination e.g.: *“People with mental illness have unpredictable behavior”;* **(ii) Stereotyping:** Four items (10-13) intended to measure the degree of respondents’ maintenance of social distance toward persons with mental illness. E.g.: *“It is easy to identify those who have a mental illness.”;* **(iii) Restrictiveness:** Four items (14-17), that hold an uncertain view on the rights of people with mental illness. E.g.: *“It is not appropriate for a person with mental illness to get married”*; **(iv**) **Benevolence (reverse coded):** Eight items (18-23, 25 and 26) related to kindness and sympathetic views of the respondents toward people with mental illness e.g.: *“People with mental illness can hold a job”;* **(v) Pessimistic prediction:** Four items (27-30) intended to measure the level of prejudice toward mental illness e.g.: *“It is harder for those who have a mental illness to receive the same pay for the same job”,* and **(vi) Stigmatization-** Four items (31-34) that measure the discriminatory behavior of the nurses toward mental illness. For example; *“Mental illness is a punishment for doing some bad things”.* The above described tools were translated and back translated into regional language (Kannada).

### Attitude scale for mental illness

Data collection Procedure. Data was collected from nurses and midwives just before commencement of a training program on mental illness at National Institute of Mental Health and Neurosciences, Bangalore. On introduction, the primary author explained briefly about aims and methods of the present study to all the subjects. Subjects who were willing to participate were asked to complete the Kannada version of the questionnaires. It took approximately 20 minutes to complete the questionnaires. 

Ethical consideration. The research protocol was reviewed and approved by the Institute Ethics Committee. Subjects were explained briefly about aims and objectives of the study and requested to participate in the study. Written informed consent was obtained from all the subjects and they were given freedom to withdraw from the study at any time. Confidentiality of the subjects was assured as the data collection tools did not include any identifying information (such as name, address, mobile number, etc,).

Data analysis. The negatively worded items (Benevolence domain) were reverse coded before the analysis. The data were analyzed using appropriate statistical software (SPSS 21 version) and results were summarized and presented in the form of tables. Independent *t* test was performed to examine whether significant differences existed between attitude scores and socio-demographic variables. Statistical significance was considered at *p*<0.05.

## Results

The mean age of the respondents was 37.90±10.60 (M±SD). More number of the subjects (37.3%) were below 30 years old, women (79.4%), Hindus (92.1%) and belonged to rural background (69.8%). Nearly half of the subjects (46.8%) were working as Auxiliary Nurse Midwives -ANM-. A majority of the subjects (54%) had less than 10 years of experience and were interested to learn about mental illness (66.6%) (Table1).

**Table 1:** Description of the sample

*Variable*	*Frequency*	*Percentage*
*Age (years)*		
*<30*	47	37.3
*31-40*	26	20.6
*41-50*	26	20.6
*>51*	27	21.4
*Gender*		
*Male*	26	20.6
*Female*	100	79.4
*Religion*		
*Hindu*	116	92.1
*Muslim*	2	1.6
*Christian/others*	8	6.3
*Residence*		
*Rural*	88	69.8
*Urban*	38	30.2
*Education*		
*Auxiliary Nursing Midwifery*	59	46.8
*General Nursing and Midwifery*	59	46.8
*Bachelor of Science in Nursing/Others*	8	6.4
*Professional experience (years)*		
*<10*	68	54.0
*11-20*	19	15.0
*>21*	39	31.0
*Level of interest to learn about mental illness*		
*<5*	22	17.5
*6-8*	20	15.9
*>8*	84	66.6

With regard to source of information, 81.7% of the subjects agreed that they were aware about mental illness through nursing education, followed by internet (77%), professional experience (63.5%) and witnessed mental illness among friends and relatives (34.1%). While, 32.5% of the subjects got information through posters (32.5%), a small number i.e. 27% of them stated that newspapers were the source of information on mental illness.

Table 2 presents true responses of the subjects to the knowledge assessment questionnaire. The mean score was 10.88±1.58 on knowledge questionnaire that suggests 91% of the subjects had good knowledge on risk factors for developing mental illness. While majority of the subjects (95.2%) accepted that head injury and physical abuses are the main causes for developing mental illness, 27.8%, 11 % of them believed that mental illness is communicable and occurs due to fate or karma.

**Table 2:** True responses of the Subjects on knowledge assessment questionnaire about the causes of the Mental illness

Variable	Frequency	Percentage
Genetic inheritance	118	93.7
Substance/alcohol abuse	121	96
God’s punishment.	119	94.4
Brain disease	113	89.7
Personal weakness	117	92.9
Possession by evil spirits	116	92.1
Traumatic injury or head injury	120	95.2
Physical abuse	120	95.2
Poverty	118	93.7
Fate or karma	111	88.1
Communicable disease	91	72.2
Neurotransmitters	107	84.9

Table 3 describes mean scores of the various domains of the ASMI scale. Higher mean score indicates negative attitudes towards mental illness in all the domains except ‘benevolence’ domain. With regard to ‘Separatism’ domain, the mean score was 26.76 with 5.70 standard deviation indicates that merely 46.5% of the subjects had positive attitudes towards persons with mental illness. Similarly, the mean score was higher in ‘Stereotyping’ domain which indicates 73% of the subjects held negative stereotypical attitudes towards people with mental illness. More than half of the subjects (54%) were less benevolent towards persons with mental illness (21.06±8.40, M±SD). Likewise, higher mean score (10.60±4.04) was observed in ‘Pessimistic prediction’ domain that suggests that 53% of the subjects endorsed negative attitudes towards persons with mental illness in this domain. However, less than cut-off point scores were observed in restrictiveness (8.79, 88%) and Stigmatization (7.18, 72%) domains. The overall mean score (88.94±13.66) reports that majority (52%) of the subjects had negative attitudes towards people with mental illness.

**Table 3:** Mean scores of responses of the participants to the attitude towards mental illness scale

Subscales	No. of items	Possible score	Cut-off (mid) point	Mean	Standard deviation
Separatism	10	5-50	25	26.76	5.70
Stereotyping	4	4-20	10	14.55	3.70
Restrictiveness	4	4-20	10	8.79	4.13
Benevolence	8	8-40	20	21.06	8.40
Pessimistic prediction	4	4-20	10	10.60	4.04
Stigmatization	4	4-20	10	7.18	3.49
Total score	34	34-170	85	88.94	13.66

Independent t test reveals that age, residence, religion and professional experience were not significantly associated with knowledge and attitudes towards mental illness among the respondents. While gender differences were not observed with regard to knowledge level of the subjects, the attitudes were significantly differed in ‘Stereotyping’ *(t*=3.462, *p*<0.001), ‘Restrictiveness’ *(t*=3.182, *p*<0.01), ‘Benevolence’ (*t*=4.10, *p*<0.001) and ‘Pessimistic prediction’ (*t*=2.22, *p*<0.05) domains as the mean score of the women were lesser compared to men indicating women held positive attitudes towards persons with mental illness. The subjects with higher professional qualification were found to have adequate knowledge as the mean score was higher than ANMs (11.33±1.12) and significant difference was noted (*t*=-3.53, *p*<0.01). None the less ANMs were found to be less restrictive (*t*=-2.96, *p*<0.05), more benevolent (*t*=-7.31, *p*<0.001) and less pessimistic *(t*=-2.69, *p*<0.05) when compared to the subjects with higher qualification in nursing. Further, the subjects who were more benevolent (*t*=2.25, *p*<0.05) and had less stigmatic attitudes (*t*=3.28, *p*<0.001) were more interested to learn about mental illness (Table 4).

**Table 4:** Comparison of mean scores for knowledge questionnaire and Attitude scale for mental illness scales with socio demographic variables

		Knowledge	Separatism	Stereotyping	Restrictiveness	Benevolence	Pessimistic prediction	Stigmatization
Age	Mean±SD	10.90±1.59	26.77±5.43	14.93±3.64	8.75±4.11	27.40±8.51	10.85±3.56	7.03±3.10
**<40 (*n*=73)**	Mean±SD	10.85±1.59	26.75±6.11	14.02±3.74	8.83±4.19	26.23±8.26	10.26±4.65	7.40±3.99
**≥40 (*n*=53)**	*t*-value	0.19	0.01	1.37	-0.10	0.77	0.80	-.58
Gender	Mean±SD	11.38±0.80	28.04±5.30	16.69±2.01	11.00±3.88	32.58±3.31	12.15±3.90	7.58±3.38
**Male (*n*=26)**	Mean±SD	10.75±1.71	26.43±5.78	13.99±3.84	8.21±4.01	25.43±8.69	10.20±4.01	7.08±3.53
**Female (*n*=100)**	*t*-value	1.83	1.28	3.46***	3.18**	4.10***	2.22*	0.64
Religion	Mean±SD	10.90±1.61	26.74±5.73	14.68±3.58	8.80±4.19	26.91±8.50	10.40±4.03	7.06±3.22
**Hindus (*n*=116)**	Mean±SD	10.70±1.34	27.00±5.62	13.00±4.81	8.60±3.47	26.80±7.38	12.90±3.63	8.60±5.87
**Others (*n*=10)**	*t*-value	0.37	-0.137	1.38	0.14	0.04	-1.89	-1.34
Professional qualification	Mean±SD	10.37±1.87	25.92±6.08	13.49±3.92	7.66±3.73	22.02±9.19	9.59±4.24	7.00±3.90
**ANMs (*n*=59)**	Mean±SD	11.33±1.12	27.50±5.28	15.48±3.24	9.78±4.23	31.21±4.36	11.49±3.67	7.34±3.12
**Others (*n*=67)**	*t*-value	-3.53**	-1.57	-3.11*	-2.96*	-7.31***	-2.69*	-.549
Residence	Mean±SD	10.82±1.56	26.61±6.14	14.59±3.79	8.86±4.37	26.35±8.98	10.24±4.19	7.39±3.67
**Rural (*n*=88)**	Mean±SD	11.03±1.65	27.10±4.58	14.45±3.52	8.60±3.53	28.18±6.77	11.45±3.61	6.71±3.49
**Urban (*n*=38)**	*t*-value	-0.67	-0.44	0.19	0.32	-1.13	-1.55	0.99
Professional experience (Yrs)	Mean±SD	11.00±1.47	26.79±5.27	15.10±3.67	8.94±4.16	28.06±8.29	11.07±3.78	7.34±3.48
**<10 (*n*=68)**	Mean±SD	10.74±1.72	26.72±6.22	13.90±3.65	8.60±4.11	25.55±8.38	10.05±4.30	7.00±3.53
**≥10 (*n*=58)**	*t*-value	0.91	0.07	1.84	0.46	1.68	1.42	0.54
Level of interest	Mean±SD	11.18±1.14	28.82±5.85	15.73±1.80	9.41±3.28	30.50±4.23	11.18±4.34	9.32±4.08
**<8 (*n*=22)**	Mean±SD	10.82±1.66	26.33±5.60	14.30±3.95	8.65±4.29	26.14±8.86	10.48±4.00	6.73±3.20
**≥8 (*n*=104)**	*t*-value	0.98	1.88	1.66	0.78	2.25*	0.74	3.28**

## Discussion

To best of our knowledge, this was the first study from India that examined primary care nurses’ attitudes towards mental illness using internationally standardized scale. The findings revealed that though the subjects had adequate knowledge on etiology of the mental illness, they possessed negative attitudes towards people with mental illness in various sub domains. 

In line with previous research, our study sample also mainly comprised of women and Hindus.(17) Our findings revealed that younger nurses were more interested to learn about mental illness. These findings may be an important concern because with adequate training and support, they can help individuals with mental illness in providing high quality of care in the community.

With regard to source of information on mental illness, 81.7% of them agreed that they learnt about mental illness through nursing education and internet (77%). These findings were dissimilar to a study from Australia, which found that TV was the main source of information for the general population.(18) However, in case of nursing professionals, nursing education at all the levels includes mental illness as part of curriculum in India. Yet in the present study, few subjects felt that they received information on mental illness through Television/Movies (31%), News papers (27%) and posters (32.5%). Mass media has a strong influence in shaping the attitudes of individuals towards persons with mental illness. Hence, governmental and non-governmental agencies as well as stakeholders need to be proactive in disseminating information on mental illness and portraying positively about persons with mental illness. The mean score 10.88(1.58, SD) on knowledge questionnaire suggests that 91% of the subjects in the present study were found to have adequate knowledge on etiology of mental illness. While these findings were supported by earlier research conducted among nurses,(19) on the contrary, only 50% of nurses from Ethiopia had adequate knowledge.(20) Yet, it cannot be ignored that 27.8% and 11 % of the nurses in the current study, believed that mental illness is a communicable disease and mental illness occurs due to fate or karma. Therefore, the educational programs should aim at changing prejudice and negative beliefs. These findings were comparable to a study conducted in Nepal(19) None the less, merely 2% and 4% of the nurses believed that mental illness is contagious and due to own sin. 

In the present study, less than half (46.5%) of the subjects held positive attitudes towards persons with mental illness as the mean score was higher than average (26.76, 5.70 SD) in this domain. These findings though similar to a study among Indian nursing students, environment,(9) were not supported by a study on nurses in South Africa.(17) Similarly, majority (73%) of the subjects held negative stereotypical attitudes towards people with mental illness. This number is higher comparing to earlier research conducted in India and elsewhere.(9,17) The findings also revealed that more than half the subjects (54%) endorsed less benevolent and more pessimistic (53%) attitudes towards persons with mental illness. These mean scores were lesser comparing to other studies using the same scale. (9,17) Never the less, 88% and 72% of the nurses hold less restrictive and less stigmatized attitudes towards mental illness. Though, these findings were almost similar to other studies, (9,17) it should not be ignored that 28% of the nurses had stigmatizing attitudes towards individuals with mental illness. However, the total mean score on attitude questionnaire revealed that more than half of the nurses (52%) had negative attitudes towards mental illness which needs an urgent attention. These findings were in concordance with earlier research carried out among nurses. (5,21,22) On the contrary, several other studies also observed nurses having positive attitudes towards persons with mental illness.(13,23,24) The negative attitudes among nurses may not only lead to discrimination,(25) but also worsen the symptoms which can affect the chances of recovery of the people with mental illness. Hence, nurses need to be empowered with adequate knowledge and proper guidelines with basic principles of mental health care.

Contrary to our findings, it was well documented that older age was significantly associated with positive attitudes towards mental illness.(2,5) However, a recent Indian study reported that younger nurses had positive attitudes towards mental illness.(13)Similar to earlier studies(3) our study also noted that women hold positive attitudes towards persons with mental illness. They were found to be less stereotypy, less restrictive and more benevolent towards people with mental illness. These findings contradict a study that reported more positive attitudes among men than women in all subscales, except for benevolence.^(13)^ Although, nurses with higher education were found to have better knowledge than ANMs. ANMs had positive attitudes in all the subscales except ‘Stigmatization’ domain. These findings could be due to their contact with people with mental illness in their professional practice. However, these findings were not in favor of earlier research that found a strong association between lower level of nursing education, social discrimination and social restriction.(4) Nurses who were more benevolent and with less stigmatized attitudes were more interested to learn about mental illness.

Strengths and limitations. The main strengths of the present study include random sample which can be representative of Karnataka state, using internationally standardized scale and with good response rate (77.8%). The present study was not an exception to certain limitations such as cross sectional descriptive study, small sample size and the data was collected using self reported questionnaires. Hence, our findings may not be representative of the nurses across the country which makes it difficult to generalize the findings. Further, it is necessary to consider the responses of the nurses with caution as there may be possibility of social desirability in their responses. Despite the limitations of the study, we emphasize that our findings offer relatively significant findings about primary care nurses’ attitudes towards people with mental illness. Therefore, our findings may be helpful in developing academic programs to tackle the negative attitudes of nurses towards people with mental illness.

## Conclusion

The present study showed that primary care nurses had adequate knowledge on mental illness. Yet it is an urgent concern to note that stigmatizing and negative attitudes prevail among primary care nurses which could affect the quality of care they provide to the people with mental illness. Moreover, it may seriously impact detection, referral and support for the individuals with mental illness in a developing country with few resources where de-institutionalization of people with mental illness is still in rudimentary stage. While the main emphasis is on integration of mental health in to primary care, it is a serious concern to inculcate positive attitudes among nurses through appropriate training programs on mental illness.
